# Effect of Incubation on Bacterial Communities of Eggshells in a Temperate Bird, the Eurasian Magpie (*Pica pica*)

**DOI:** 10.1371/journal.pone.0103959

**Published:** 2014-08-04

**Authors:** Won Young Lee, Mincheol Kim, Piotr G. Jablonski, Jae Chun Choe, Sang-im Lee

**Affiliations:** 1 Laboratory of Behavioral Ecology and Evolution, Department of Biological Sciences, College of Natural Sciences, Seoul National University, Seoul, Republic of Korea; 2 Laboratory of Prokaryotic Biology and Bioinformatics, Department of Biological Sciences, College of Natural Sciences, Seoul National University, Seoul, Republic of Korea; 3 Museum and Institute of Zoology, Polish Academy of Sciences, Warsaw, Poland; 4 Division of EcoScience, Ewha Womans University, Seoul, Republic of Korea; 5 Institute of Advanced Machinery and Design, Seoul National University, Seoul, Republic of Korea; University of Akron, United States of America

## Abstract

Inhibitory effect of incubation on microbial growth has extensively been studied in wild bird populations using culture-based methods and conflicting results exist on whether incubation selectively affects the growth of microbes on the egg surface. In this study, we employed culture-independent methods, quantitative PCR and 16S rRNA gene pyrosequencing, to elucidate the effect of incubation on the bacterial abundance and bacterial community composition on the eggshells of the Eurasian Magpie (*Pica pica*). We found that total bacterial abundance increased and diversity decreased on incubated eggs while there were no changes on non-incubated eggs. Interestingly, Gram-positive *Bacillus*, which include mostly harmless species, became dominant and genus *Pseudomonas*, which include opportunistic avian egg pathogens, were significantly reduced after incubation. These results suggest that avian incubation in temperate regions may promote the growth of harmless (or benevolent) bacteria and suppress the growth of pathogenic bacterial taxa and consequently reduce the diversity of microbes on the egg surface. We hypothesize that this may occur due to difference in sensitivity to dehydration on the egg surface among microbes, combined with the introduction of *Bacillus* from bird feathers and due to the presence of antibiotics that certain bacteria produce.

## Introduction

Nest building and egg incubation behaviors of birds are well known as adaptations to provide favorable conditions for growth and survival of offspring [1], [2]. It has also been discovered that parents, by using specific nest materials, are able to control the abundance and composition of parasite [3]–[5] and bacterial load [6] in the nests in an adaptive manner. Particularly, controlling the abundance and composition of the bacterial communities on the eggshells would be important because bacterial infections through the eggshell decrease egg viability [7]–[11]. Therefore, any defense mechanism against proliferation of pathogenic microbes on the egg surface would be advantageous. Recent research suggested that incubation affects bacterial communities on eggshells [12], [13], and several mechanisms are proposed for this effect. Parental incubation may affect microbial communities on eggshells by inhibiting total growth of bacteria through making eggshell conditions more adverse for microbial growth [12], [14], [15] or by promoting the presence of antibiotic-producing bacteria originating from bird feathers and causing competition-mediated changes of abundance and composition in bacterial communities on the eggshells [13] or oil glands [16].

The effect of incubation on the bacterial loads on eggshells in the tropical environment was studied using both culture-dependent [10], [11] and culture-independent [12] methods and the results differed. In a study that employed the culture method, Cook et al. [10], [11] found that, although overall abundance of microbes decreased, the abundance of pathogenic bacteria decreased and that of harmless bacteria (Gram-positive rods) increased with incubation indicating that changes of bacterial community during incubation lead to communities that are beneficial to the birds. However, in a study that employed culture-independent method on the same bird species, Shawkey et al. [12] found that both microbial abundance and diversity were inhibited by incubation and they did not find any evidence of the selective proliferation of harmless bacteria by incubation. The reason why the two studies on the same species and the same study population found somewhat different results remains unclear; it might be due to methodological difference (between culture-dependent techniques [10], [11] and culture-independent techniques [12]), or difference in conditions between the sampled years (2002 for Cook et al.'s study [10] and 2007 for Shawkey et al.'s study [12]).

In contrast to the wet tropical environment where incubation seems to dry the eggshell and the proliferation of microbes on the egg surface is generally suppressed [12], recent studies conducted in the temperate environment produced conflicting results. Whereas some found that incubation decreases total bacterial abundance on the eggshells [14], [15], others failed to find significant changes in bacterial loads on the incubated eggs [17], [18]. Therefore, it is still unclear whether incubation in the temperate environment inhibits microbial communities on the eggshells as it does in the tropics.

Nearly all of the previous studies on bacterial loads on the eggs have used culture-based methods [10], [11], [15], [18]–[20]. Culture-based quantification of microbes has its own merits such as easiness to implement and low cost. However, there can be problems with left censoring in counting the colony numbers (briefly reviewed in [21]) and the method can only provide data on the abundance of a few specific target microbes because less than 1% of microbial species are culturable [22]. Therefore most of the previous studies did not provide complete information about effect of incubation on the abundance and diversity at a level of microbial community. Particularly, no study of birds from the temperate region used culture-independent methods.

Here, we employed culture-independent methods to examine the effect of incubation on the structure of microbial community on the eggshells. First, we conducted quantitative PCR (qPCR), which is known to be more sensitive than culture-based method [23], [24], to estimate total bacterial abundance. Second, we performed pyrosequencing of the 16S rRNA gene to examine diversity and relative abundance of the various microbe taxa on the eggshells. NGS (next generation sequencing) approaches, including pyrosequencing, allow researchers to find microbial community structures by reading 16S rRNA gene sequences of bacteria from complex samples [25]. Among other culture-independent methods (e.g. microarrays in [12]), pyrosequencing technology has been a powerful tool to study microbial samples since this method is suitable to estimate unknown microbial community by searching for bacteria-specific sequences in 16S rRNA (for review see Lee et al. [26]).

In this study, we tested if avian incubation affects the eggshell bacterial community in a temperate zone environment with the Eurasian Magpie (*Pica pica*) and discussed possible mechanisms based on our results.

## Results

**Figure 1:**
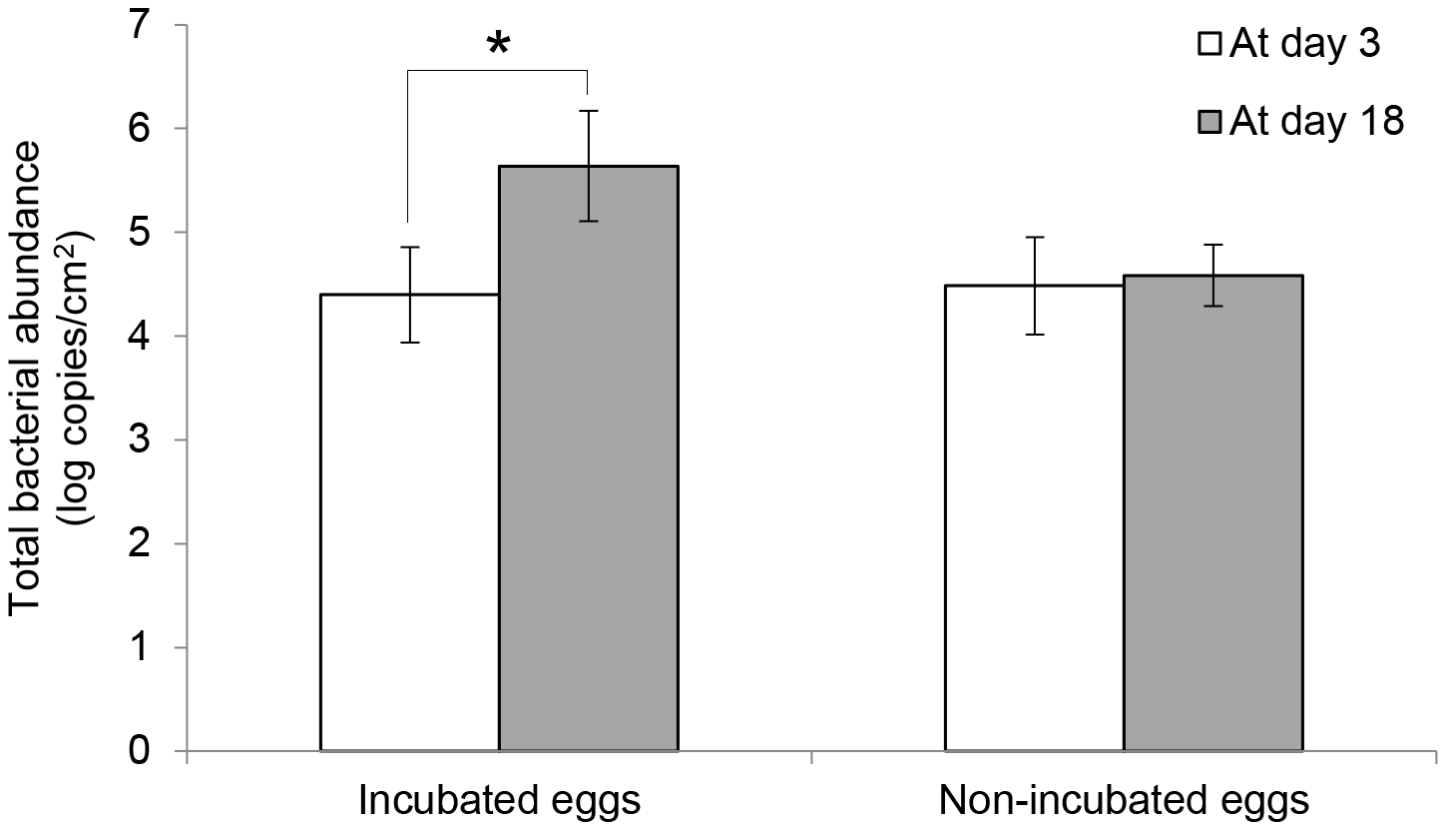
Changes in the microbial abundance on incubated and non-incubated eggs. Total bacterial abundance was estimated with log-copy numbers per square centimeter obtained from quantitative PCR with a bacterial universal primer set (333F-518R) in 9 incubated nests and 3 control nests at day 3 (before incubation) and day 18 (after incubation). Asterisks represent significant difference (*P*<0.05) by paired t-test between day 3 and day 18. Error bars denote standard errors.

Total bacterial abundance, estimated by qPCR for microbial 16S rRNA gene, on naturally incubated magpie eggs increased (approximately 26 times on average) during the 15 days of incubation period, whereas there was no significant increase during the same duration on non-incubated control eggs (Figure 1; Paired t-tests, *T* = −2.67, *DF* = 8, *P* = 0.03 on incubated eggs and *T* = −0.17, *DF* = 2, *P* = 0.88 on non-incubated eggs).

Using pyrosequencing method, we detected a total of 4434 OTUs (operational taxonomic units) that includes 1590 genera, 553 families, 221 orders, 90 classes and 32 phyla of bacteria (Table S1. There were no differences in the initial bacterial community structure between the non-incubated control eggs (sampled 3 days after the artificial nest has been setup) and the incubated eggs (sampled at day 3 after egg-laying) (ANOSIM, *R* = 0.22, *P* = 0.84), which confirms that, as intended, the initial bacterial communities did not differ statistically between control and incubation treatments.

**Figure 2:**
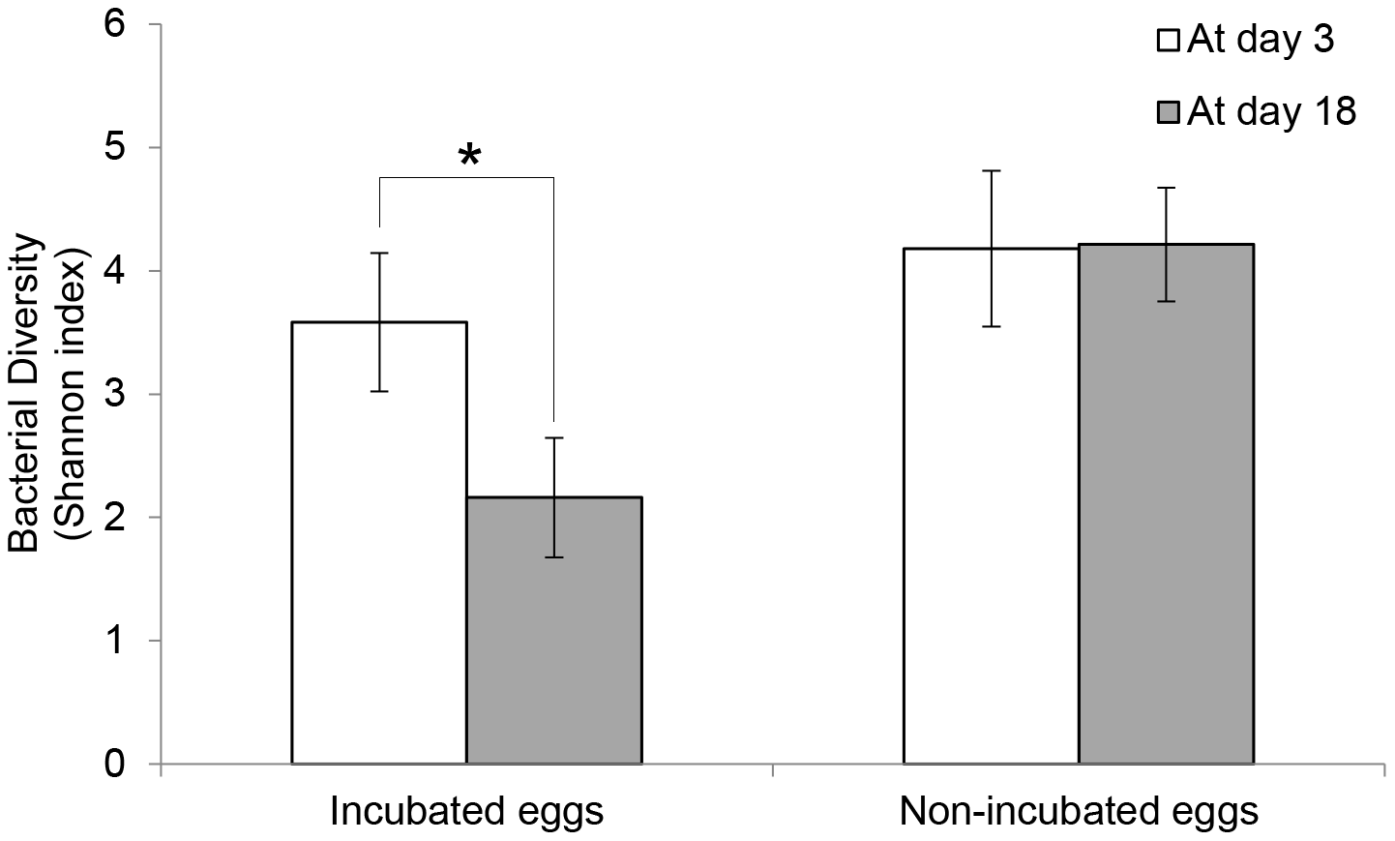
Bacterial diversity (Shannon index; mean) of incubated and non-incubated eggs. Bacterial diversity was estimated by Shannon index with 570 sequences per sample acquired from 16S rRNA gene pyrosequencing, at day 3 and day 18 after egg laying in 9 incubated nests and 3 control nests. Asterisks represent significant difference (*P*<0.05) by paired t-test between at day 3 and day 18. Error bars denote standard errors.

**Figure 3:**
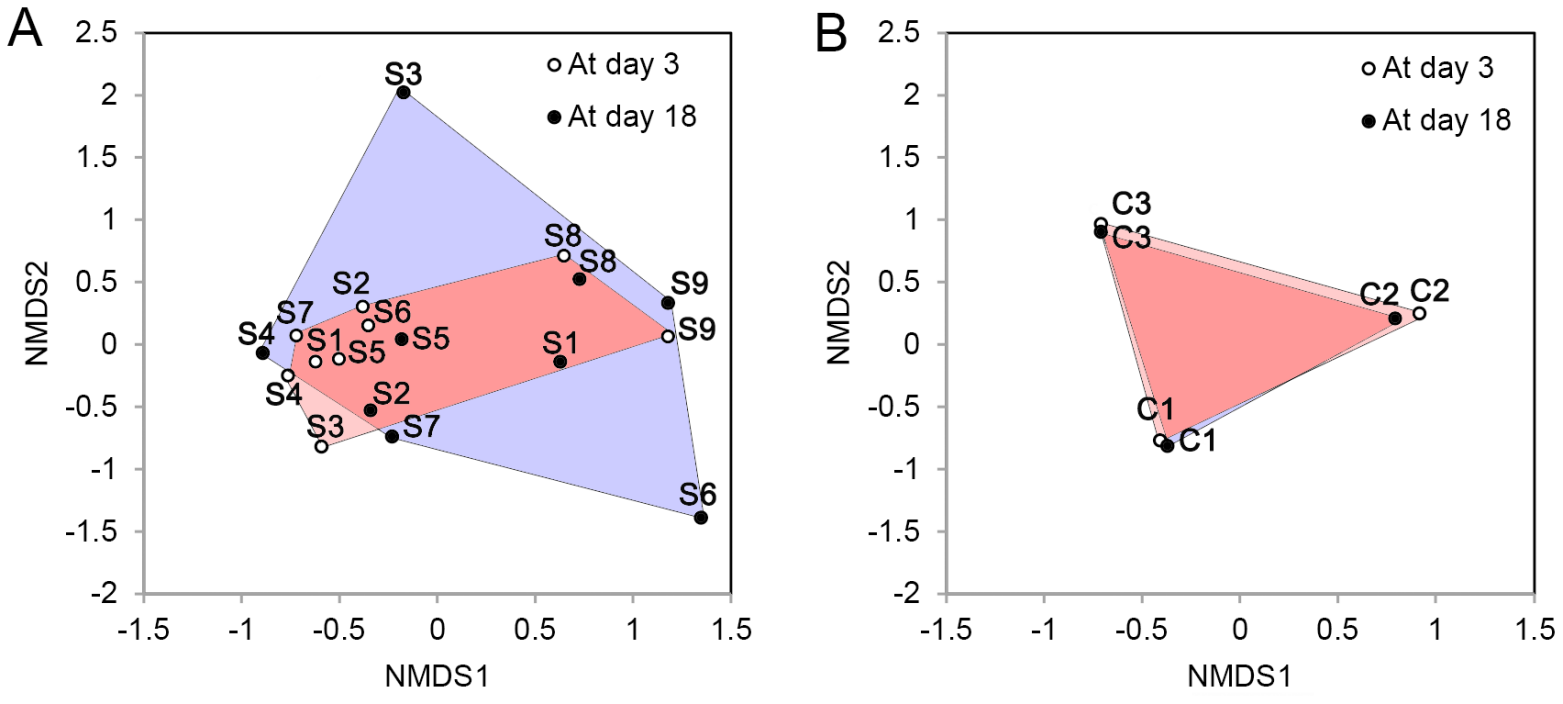
NMDS plots of bacterial community structure at day 3 and day 18 after the first egg was laid, in 9 incubated nests (A) and three control nests (B). NMDS (Nonmetric Multidimensional Scaling) plots were generated with Bray-Curtis dissimilarities between samples in 9 incubated nests, S1–S9, in (A) (2D stress  = 0.12) and 3 control nests, C1–C3, in (B) (2D stress  = 0.01). NMDS plot is mapping the bacterial structure of each sample considering the relative distances with other samples, based on both bacterial composition and abundance. Pink polygon contains all initial bacterial communities; the blue polygon encompasses all bacterial communities at the end of incubation.

Bacterial diversity (Shannon index) was significantly reduced on incubated eggs but not on the non-incubated eggs (Figure 2; Paired t-test, *T* = 2.48, *DF* = 8, *P* = 0.04 on incubated eggs and *T* = −0.13, *DF* = 2, *P* = 0.91 on non-incubated control eggs). The bacterial assemblages on incubated eggs became more dispersed and dissimilar after incubation (between day 3 and day 18 after first egg-laying; IMD = −0.50; Welch two sample t-test using Bray-Curtis dissimilarity distances, *T* = 2.74, *DF* = 5.64, *P* = 0.04; Figure 3A) whereas those on non-incubated eggs remained little changed between first and second samplings (second sampling was done 15 days after the first sampling; IMD = 0.11; Figure 3B). There was no apparent incubation-specific change in the bacterial community structures (ANOSIM; R = 0.03, p = 0.23 on incubated eggs, Figure 3A; *R* = −0.41, *P* = 0.90 on control eggs, Figure 3B): composition in some incubated nests changed little (e.g. S4, 8, 9).

Dufrene-Legendre Indicator Species Analysis identified 8 indicator bacterial taxa (genera) that are strongly associated with the composition of initial bacterial communities on the incubated eggs (at day 3 after egg laying; Table 1). The indicator taxa were mostly soil bacteria. Some those taxa also included two opportunistic pathogenic genera (*Flavobacterium* and *Acinetobacter*) and one genus of antibiotic-producing bacteria (*Streptomyces*). However, no indicator taxa were found after 15 days of incubation (at day 18 after egg laying). These results indicate that incubation does not uniformly affect the taxonomic composition of the community across all nests, but it rather creates variable eggshell communities (from the taxonomic composition point of view).

**Table 1:** The result of Dufrene-Legendre Indicator Species Analysis.

Genus name	Indicator value	*P* value	Mean percentage (SE)	Characteristics
*Rhizobium*	0.67	0.021	0.91 (0.17)	Nitrogen fixing in soil [82]
*Arthrobacter*	0.67	0.042	1.18 (0.29)	Found in soil [83]
*Streptomyces*	0.56	0.031	0.24 (0.06)	Antibiotic-producing [84]; mostly found in soil and decaying vegetation [85]
*Flavobacterium*	0.56	0.036	6.11 (5.46)	Found in soil and opportunistic pathogens in fish [86]
*Chryseobacterium*	0.56	0.030	0.14 (0.05)	Found in soil and plants [87]
*Variovorax*	0.55	0.047	0.83 (0.59)	Found in soil [88]
*Microlunatus*	0.55	0.035	0.16 (0.04)	Found in soil [89]
*Acinetobacter*	0.50	0.024	1.12 (0.68)	Opportunistic pathogens in human [90]; found in soil [91]

This shows 8 indicator genera on incubated eggs at day 3. The analysis did not reveal any indicator genera for samples collected at day 18.

**Figure 4:**
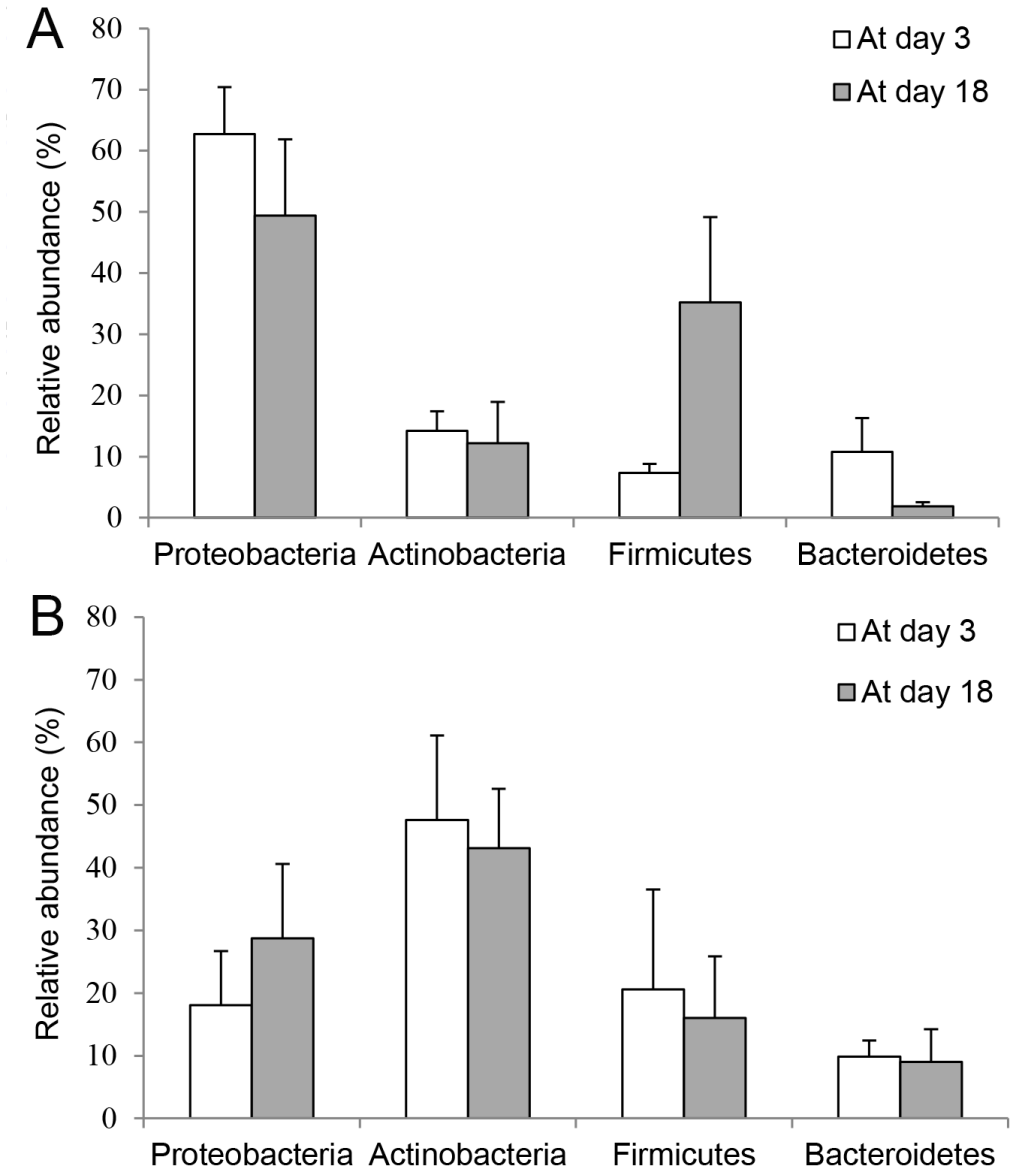
Relative abundance of four major bacterial phyla on eggshells at day 3 and day 18, in 9 incubated nests (A) and three control nests (B). Relative abundance (%, +SE) was obtained from 16s rRNA gene pyrosequencing, and that of four major bacterial phyla, among 32,accounted for more than 90% of sequences in 9 incubated nests (A) and 3 control nests (B) at day 3 and day 18.

**Figure 5:**
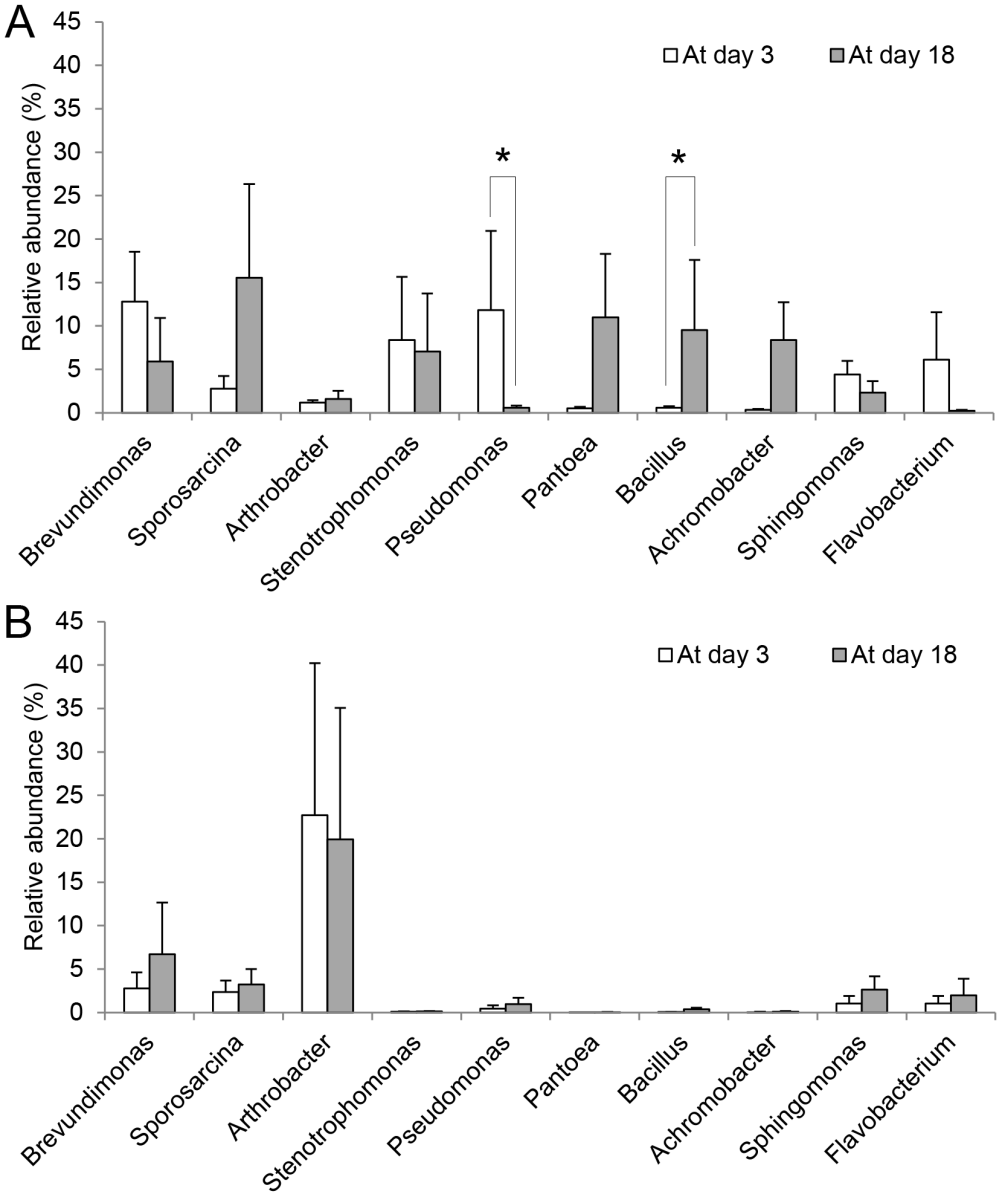
Relative abundance of 10 most abundant bacterial genera on eggshells at day 3 and day 18, in 9 incubated nests (A) and 3 control nests (B). Relative abundances (%, mean +SE) of 10 major bacterial genera, among 1590, accounted for more than 50% of sequences in 9 incubated nests (A) and 3 control nests (B) at day 3 and day 18. Asteriks represent significant difference (*P*<0.05) between at day 3 and day 18 by paired t-tests.

Relative abundance of four most abundant bacterial phyla (comprising more than 90% of all sequences) is shown in Figure 4, and that of 10 most abundant bacterial genera (comprising more than 50% of all sequences) is given in Figure 5. At the phylum level, we did not find any significant differences in changes of microbial abundance between incubated and non-incubated eggs (Repeated measures MANOVA: Wilk's lambda  = 0.78, *F* (4, 7) = 0.50, *P* = 0.74). However, at the genus level, the changes in microbe abundance between incubated and non-incubated eggs was marginally significant (Wilk's lambda <0.01, *F* (10, 1) = 214.01, *P* = 0.05). Paired t-tests for comparison of relative abundance between at day 3 and 18 on each genus separately revealed that relative abundance of *Pseudomonas* significantly decreased (Paired t-test, *T* = 2.99, *DF* = 8, *P* = 0.02) whereas that of *Bacillus* increased (*T* = −2.71, *DF* = 8, *P* = 0.03) on incubated eggs. We further attempted to identify the species using EzTaxon-e database with more than 99% of 16S rRNA gene sequence similarities. We identified two pathogenic *Pseudomonas* spp., *P. oryzihabitans* (GenBank accession no. D84004) and *P. plecoglossicida* (accession no. AB009457), and three antibiotics-producing *Bacillus* spp., *B. subtilis* (accession no. ABQL01000001 and EU138467), *B. pumilus* (accession no. ABRX01000007), and *B. licheniformis* (accession no. AE017333).

## Discussion

We found that temperate bird eggshells harbor a diversity of bacterial taxa, perhaps even more diverse than those found by Shawkey et al. [12] on tropical bird eggshells (553 families and 221 orders on magpie eggs in our study using pyrosequencing; 256 families and 138 orders on thrasher eggs in Shawkey et al. [12] using microarray-based PhyloChip analysis). Although pyrosequencing is more commonly used than microarray-based estimation on microbial community due to its cost efficiency and sensitivity [27], recent studies comparing the two methods provided strong correlations in terms of diversity and relative abundance [28]–[30].

Previous studies suggested that both, eggshell bacterial abundance and diversity, decrease (or at least do not increase) during incubation [10]–[12], [15], [19]. In contrast, we found that the total microbial abundance increased and microbial diversity decreased after incubation. This corresponds to the results of a recent study [31] conducted in a temperate area on pigeon eggshells. Grizard et al.’ study [31], which employed similar methods as our study, showed that bacterial abundance increased while diversity decreased and they observed a selective growth pattern of specific bacterial taxa. However, they did not examine the composition of microbial community on the non-incubated eggs; thus the result might not be directly related to the effect of incubation. Through comparisons of changes in microbial communities between non-incubated eggs and incubated eggs, we obtained similar results as Grizard et al. that some bacterial taxa became dominant on the eggshells at the expense of others. Moreover, we found that many of these increasing/decreasing taxa might have been different in each nest (as suggested by Dufrene-Legendre Indicator Species Analysis and the NMDS scattergrams, Figure 3A). Dufrene-Legendre analysis revealed that 8 indicator taxa that are characteristic for the initial phase of incubation (day 3), include both pathogenic (e.g. *Flavobacterium* and *Acinetobacter*) and non-pathogenic (e.g. *Arthrobacter*, *Variovorax*, and *Microlunatus*) bacteria that were mostly derived from soil, which is not surprising considering that magpie nests contain a layer of locally collected soil material [32] and that magpies forage on ground [32], [33]. However, no indicator taxa were detected on the eggs that were naturally incubated for 15 days. This suggests that incubation caused an average eggshell bacterial assemblage not only to be more abundant and less diverse, but it also promoted divergence among the communities in different nests.

There were no significant differences in eggshell bacterial communities between incubated magpie eggs and control quail eggs at day 3. As the surface of control quail eggs was sterilized three days prior to the first sampling, the microbes that were present on the egg surface at the first sampling have most likely originated from the natural nest materials that were put into the artificial nests. It seems that three days of exposure to nest materials that have been taken out of deserted nests (see Methods for details) are sufficient for the eggshell to form the bacterial communities similar to the eggs that were naturally in the nests. This result also implies that most of the eggshell microbes may have originated from the nest materials rather than from mothers' bodies. Although magpie eggshell may harbor the microbes that originate from maternal gut or cloaca, it seems that the influence of these microbes on the microbial community structure is rather small. In addition, microbial community on the eggshell of the control nests did not change much after 15 days of incubation whereas that of incubated nests went through substantial changes. This clearly shows that the changes in microbial community structure on the eggshell throughout 15 days of incubation could be solely attributed to the incubation by females.

Despite the divergence in bacterial community composition among nests during incubation, there were several common patterns suggesting a presence of some universal effects of incubation. Two out of 10 most abundant genera showed significant changes in abundance in almost all incubated nests: *Pseudomonas* decreased and *Bacillus* increased. *Pseudomonas* includes opportunistic human pathogens, such as *P. aeruginosa* [34] and *P. oryzihabitans* [35], [36] and a fish pathogen, *P. plecoglossicida* [37]. On the other hand, the majority of *Bacillus* are harmless [38] and some are reported to produce antibiotics (e.g. *B. pumilus, B. brevis, B. subtilis, B. firmus, B. licheniformis, B. mycoides, and B. circulars*; [39]–[41]). Some of the sequences that were obtained seemed to match those of antibiotic-producing *Bacillus* (*B. sutilis, B. pumilus*, and *B. licheniformis*). Thus, our results suggest that incubation causes a decrease of potentially pathogenic bacteria that is associated with an increase of non-pathogenic or antibiotic-producing bacteria.

The selective effect of incubation on the changes in the abundance of two specific genera (*Bacillus* and *Pseudomonas*), as well as the decrease in microbial diversity and increase in total microbial abundance, can result from three interacting mechanisms. First, the incubating bird may directly increase the presence and abundance of some antibiotic-producing (or bacteriocin-producing bacteria) by transferring them from bird's body or feathers to the eggshell, or by transferring the antibiotics produced by bacteria on feathers to the eggshell. For instance, if *Bacillus* spp., known to be present on bird feathers [42], [43], produced antibiotics that suppress other bacteria, including pathogenic ones such as *Pseudomonas* spp. [44], this could explain the compositional change in eggshell microbes that was observed in our study. The antibiotics produced by *Bacillus* [45] have already been proposed as a defense mechanism against pathogens in birds [46]. Hence, if the antibiotics from *Bacillus* negatively affected other microbes, both pathogenic and non-pathogenic, it could result in lower microbial diversity after incubation. The observed relationship between increase of total abundance and decrease of community diversity is consistent with the classical theory of species diversity [47] and with modern understanding of competitive interactions in microbial communities [48], [49] when some quickly growing species with higher competitive abilities (e.g. production of antibiotics) may eliminate other species leading to a decrease in community diversity. Total increase in abundance in this case may be hypothetically promoted by a removal of negative competition-mediated effects on population growth of the remaining species in the community. Similar mechanisms based on negative competition-mediated effects have already been documented in bacterial communities where high diversity and competition among diverse taxa lead to a collapse in total abundance [50].

We also detected *Enterococcus* spp., another bacteria that is known to produce bacteriocins and antibiotics and had been isolated from uropygial glands of hoopoes [51], [52], but they were found only in three naturally incubated nests and the relative abundance was very low (0.20%±0.18; mean ± SE). Thus it is unlikely that this genus could have played a major role in changes in bacterial composition on the eggshells.

Second contributing factor may be competition among bacterial taxa through competitive use of resources (scramble competition). If incubation causes higher abundance of harmless bacteria, e.g. by supplying bacteria onto the eggshell from external sources, these bacterial may dominate the eggshell bacterial community and negatively affect the growth of pathogenic bacteria by removing vital resources from the habitat. For instance, *Bacillus* and *Pseudomonas* were reported to be competitive with each other for nitrate and glucose [53], [54]. This bacterial antagonism might cause changes in the eggshell bacterial community throughout incubation. Even if the majority of *Bacillus* that we detected in our study were not the ones that produce antibiotics, the competition for resources between these two bacteria might explain the genus-specific growth patterns (increase of *Bacillus* and decrease of *Pseudomonas*).

Third hypothetical mechanism is not based on the introduction of *Bacillus* into the eggshell bacterial community from external sources, but it relies on the differences between bacteria in their sensitivity to the humidity changes on the eggshell caused by incubation. Eggshell surface becomes dry with incubation, which previous studies, conducted both in tropics [12] and temperate regions [14], [15], suggested as the important function of incubation (“drying mechanism”). If *Bacillus* and *Pseudomonas* differ in the sensitivity to the changes in humidity on the eggshell, this may cause the differential growth between these two taxa throughout incubation. A comparison on the minimum water activity level for growth of *Bacillus subtilis* (0.91) [55] and *Psuedomonas aeruginosa* (0.97) [56] suggests that the growth of *Psedomonas* may indeed be more hampered by desiccation than that of *Bacillus*. Similar to our results, previous studies also noticed that *Pseudomonads* decrease after incubation [9], [15]. If this hypothesis is correct, then our results suggest that, unlike in the tropical region where the reduction of humidity negatively affects eggshell bacterial taxa in general [12], [15], in the temperate region the reduced humidity due to incubation may promote proliferation of some selected microbes on the egg surface.

Two out of the three hypothetical mechanisms suggest that magpies introduce *Bacillus* to the egg surface during incubation. There are three potential sources for *Bacillus*. The first is uropygial gland. Secretions from uropygial gland contain microbes that produce antibiotics as well as waxes [16], [46]. The best studied example is the strains of *Enterococcus faecalis* from uropygial glands of hoopoes *Upupa epops* [51]. However, so far there is no report of any species harboring *Bacillus* in the uropygial gland. In fact, antibioctics that are produced by *Enterococcus* are known to work against other microbes including *Bacillus* [52]. This does not seem to be confined to hoopoes, as it is known that secretions from uropygial gland inhibit *Bacillus* in other species as well (e.g. barn swallows and house finches) [57], [58]. Thus, it seems not plausible that *Bacillus* that increase with incubation originated from uropygial gland in magpies. The second possible source of *Bacillus* is the brood patch of the female. However, currently there is no evidence that avian brood patch contains special microbes. The third and the most likely possibility is the feathers. *Bacillus* represents microbes that are derived from soil and some of them are well-known as feather-degrading bacteria (e.g. *Bacillus licheniformis*) [59]. Magpies use feathers as the nest lining material and it is plausible that these feathers may serve as the source for increased *Bacillus* on the eggshell. Considering that white feathers may harbor higher density or growth of feather-degrading bacteria than dark feathers [13], white belly feathers of magpies, that make direct contact with the eggs, also potentially serve as the source for *Bacillus*.

In summary, our results suggest that avian incubation in the temperate zone, unlike the tropical zone, promotes the growth of harmless or antibiotic-producing bacterial taxa on eggshells and reduces growth of pathogenic bacteria rather than inhibiting the total bacterial growth on the eggshells. The hypothetical mechanisms involve effects of incubation on the outcome of between-species interactions in a bacterial community. We propose that the effects of incubation on the eggshell bacteria can be viewed as a specific example of “ecological engineering” [60] of bacterial community composition by incubating birds. Therefore, further studies of the effect of incubation on eggshell bacterial communities in various bird species from different geographical zones will not only determine the adaptive value of incubation behavior to birds, but they may also provide a model system for the study of ecological engineering in wild animals.

## Materials and Methods

### Ethical Statement

The research has been conducted according to relevant national and international guidelines. The procedure for this study has been approved by Institutional Animal Care and Use Committee of Seoul National University (No. SNU 130621-6).

### Study population and microbial sampling

This study was conducted on the magpie population at the Seoul National University (SNU) campus (37°47′ N, 126°95′ W; Seoul, Korea). From early March in 2012, we regularly visited magpie nests to determine the laying date of the first egg (hence ‘laying date’). Incubation starts 3–4 days after the first egg has been laid and lasts 21–22 days in our study population (see Lee et al. [21]). In 2012, the laying date ranged from 14^th^ March to 8^th^ May. The average daily temperature was 13.57°C (±6.31, SD) and the relative humidity was 51.29% (±14.57, SD) throughout the incubation period.

Microbial sample from the eggshell was collected twice per nest, at day 3 (before the full incubation started) and at day 18 (4–5 days before hatching) after the first egg was laid (for naturally incubated eggs) or after the artificial nest has been set up (for control eggs). We sampled a total of 24 eggs; two eggs each from 9 incubated nests and 3 non-incubated (control) nests. At day 3, we chose one egg randomly, swabbed the surface using a sterile rayon swab (Yuhan Lab Tech Co., Korea) wetted with sterile sodium phosphate buffer (0.2 M, pH 7.2) and mark the eggs with a marker. In order to control for the difference in surface area of the eggs, we measured the dimension of the egg using the formula S = 3×L^0.771^×W^1.229^, where S is the surface area of eggs, L is length, and W is width (from Narushin [61], for details, see Soler et al. [62]). The swab was put into the 1.5 ml tube containing 500 ul of buffer solution, moved to the laboratory as soon as possible, and stored in a deep freezer (−50°C). At day 18, we chose one another egg randomly except the egg which was marked before and sampled the egg by the same method at day 3.

As the non-incubated control group, we prepared three artificial nests in April, around the middle of time of egg-laying period. We created artificial nests by putting a cup-like structure, inside small cages, made from mixtures of nest materials retrieved from three abandoned nests. As magpies use dry grass, cotton and feathers for nest lining, all these soft materials were present in the control nests as well. We then placed two quail eggs in the middle of this cup-like structure after cleaning the eggshell surfaces with bactericidal disinfectant 70% isopropyl alcohol wipes (Yuhan-Kimberly, Korea). Quail eggs were used instead of magpie eggs that were laid by the females because the breeding success, including clutch size, of the population was carefully monitored through a long-term ecological monitoring project. There was no difference in the structure of microbial communities on eggshell surfaces between quail eggs and magpie eggs before incubation (see Result section). Thus, we think that the difference between quail eggs and magpie eggs in terms of microbial community before incubation started was negligible, and that any changes in microbial community throughout incubation can be attributed to the incubation by the mothers rather than the difference in microbial community between quail eggs and magpie eggs. The artificial nests were put at a similar height of magpie nests and distributed around SNU campus. We put the quail eggs into the artificial nests after sterilization and sampled the quail eggshells twice as we did with magpie eggs (one egg at day 3 after setting up the artificial nests and the other at day 18). The average temperature and humidity between the two sampling dates were 13.93°C (±3.04, SD) and 55.33% (±18.41, SD). From the swab samples, we extracted DNA with the MoBio PowerSoil DNA kit (MoBio laboratories, Carlsbad, USA) as directed by manufacturer's instructions.

### Quantitative PCR

To assess the total bacterial abundance, quantitative PCRs were conducted using the extracted DNA from the samples with a qPCR machine (Illumina Eco, San Diego, USA) and a universal primer set for bacteria (338F: 5′-ACT CCT ACG GGA GGC AGC AG-3′, 518R: 5′-ATT ACC GCG GCT GCT GG-3′; see Fierer et al. [63]). PCR assays were performed with 5 µl of QuantiMix SYBR 2× (PhileKorea Technology, Seoul, Korea), 0.25 µl of 10 pmol/L forward and reverse primers, 3.5 µl of ddH_2_O, and 1 µl of DNA extract of total 10 µl cocktail solution. PCR thermal condition was as follows; initial polymerase activation for 10 min at 95°C, PCR cycling for 10 sec at 95°C and for 30 sec at 60°C for 40 times. The melting curve was obtained by lowering the temperature from 95°C to 55°C. To generate standard curves, we designed recombinants of 16S rRNA target gene (Eub338F-518R region) in cloning vectors. By this method, we quantified the absolute numbers of total bacteria inferred from the reliable standard curves (for details see Lee et al. [21]). First, genomic DNA was extracted (Wizard Genomic DNA Purification Kit, Promega, Madison, USA) from an *Escherichia coli* K-12 MG1655 which was kindly donated by Laboratory of Prokaryote Biology and Bioinformatics in Seoul National University. Then, the extracted DNA was amplified (GoTaq Green Master Mix, Promega, Madison, USA) with 338F-518R primers and the amplified species-specific gene fragments were inserted into cloning vectors (3003 bp, pGEM-T Easy Vector, Promega, Madison, USA) transformed in competent cells (Hit-DH5α, RBC Bioscience, Taipei, Taiwan). Recombinants of cloning vector was selected in LB (Luria-Bertani) plates with ampicillin (100 µg/ml), IPTG (isopropyl beta-D-thiogalactoside, 0.5 mM), and X-Gal (5-bromo-4-chloro-3-indolyl β-d-galactoside, 80 µg/ml). Then, plasmid mini prep kit (Hybrid-Q, GeneAll Biotechnology, Seoul, Korea) was implemented to purify the recombinant plasmid DNA and the concentration of the plasmid DNA was measured with Quant-iT PicoGreen dsDNA assay kit (Invitrogen, Carlsbad, USA) and TBS-380 Mini-Fluorometer (Turner Biosystems, Sunnyvale, USA). The corresponding DNA copy numbers were calculated using the following equation [64], [65]: 
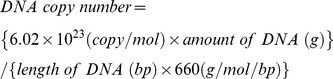



Standards were serially 10-fold diluted from 1×10^0^ to 1×10^8^ copies/µl. PCR was performed three times for each sample and the PCR results satisfied the following requirements: the efficiency of the standards were between 90% and 110%, R^2^ was higher than 0.99, and the standard deviation of PCR replicates was less than 0.167. PCR results were analysed with Eco software (version 3.0). PCR results of Ct (threshold cycle) values were converted to the absolute DNA quantity by comparing the Ct values to the serially diluted standard curves (for details see Heid et al. [66]). Melt curves were checked by increasing temperature from 55°C to 95°C every 0.5°C.

### 16S rRNA gene pyrosequencing and data processing

Extracted DNA was amplified targeting the V1 to V3 region of 16S rRNA using primers (V1-9F: 5′-Adapter A-AC-GAG TTT GAT CMT GGC TCA G-3′, V3-541R: 5′-Adapter B-barcode-AC-WTT ACC GCG GCT GCT GG-3′). The 9F primer included a 454 sequencing adapter A and a 2-bp linker sequence (AC) and the 541R incorporated a 454 adapter B, 8–10 bp barcodes specific to each sample, and a AC linker. PCR assays were performed with 0.25 µl of Pwo SuperYield DNA polymerase (Roche, Mannheim, Germany), 2 µl of 20 pmol/L forward and reverse primer, 2.5 µl of 1×BSA, 5 µl of 10×buffer, 1 µl of 10 µM dNTPs, 38.25 µl of dddH_2_O, and 1 µl of DNA extract of total 50 µl cocktail solution. PCR condition was as follows: initial denaturation for 5 min at 95°C, 10 cycles of denaturation for 30 sec at 94°C and annealing for 45 sec at 60°C to 55°C (−0.5°C per cycle) and elongation for 90 sec at 72°C for 40 times, and additional 20 cycles of denaturation for 30 sec at 94°C and annealing for 45 sec at 55°C and elongation for 90 sec at 72°C. The amplified PCR products were purified with the QIA-quick PCR purification kit (Qiagen, CA, USA) and then mixed in a pool. Pyrosequencing was performed by Macrogen Incorporation (Seoul, Korea) using 454/Roche GS-FLX Titanium Instrument (Roche, NJ, USA).

All the sequences were processed by trimming sequences to remove the unique barcode, linker, and primer sequences at both ends. In addition, we removed short sequences which were less than 300 base pairs, low-quality sequences (maximum homopolymer of 8 bp, minimum ambiguous base of 1, and minimum quality score of 25), and the sequences which had no matches to the 16S rRNA gene databases in BLASTn search (the expectation value was more than 10^−5^) to reduce sequencing error (Unno et al. 2010). The resulting sequences were further denoised using single linkage preclustering (SLP) algorithm and putative chimeras were additionally removed by using UCHIME [67] implemented in MOTHUR version 1.13.0 [68]. Quality-checked sequences were taxonomically assigned using EzTaxon-e database [69]. For species identification, conventionally sequences with ≥97% identity are considered to be from the same species [70]. Pyrosequences are available in GenBank-SRA (Accession No. SRP036169).

### Statistical methods and taxonomic analysis

We used paired t-tests to compare the total bacterial abundance (log transformed) and bacterial diversity (Shannon diversity index) at day 3 and day 18 in incubated and control nests, with SPSS version 20.0. Shannon index represents microbial diversity at community levels [71]. This was calculated based on the operational taxonomic units (OTUs), which were defined at 97% similarity cutoff of 16S rRNA gene sequence, by a randomly selected subset of 742 reads per sample in Primer v6 [72].

We used Primer v6 software [73] to estimate Bray-Curtis dissimilarity between bacterial communities at day 3 and day 18 within the nests and to generate a non-metric multidimensional scaling (NMDS) separately for naturally incubated magpie eggs and the non-incubated control eggs. We compared the paired distances within the same nests, which were from Bray-Curtis dissimilarities, between incubated and control nests by Welch two sample t-test in R [73]. Additionally, we used Index of Multivariate Dispersion (IMD) using multivariate dispersion indices (MVDISP) from Bray-Curtis dissimilarity matrix, to evaluate the relative variability between groups [74] using Primer v6 software. The IMD value, which varies from −1 to +1, indicates a relative score of differences between the variability of different groups. The IMD values of zero means no difference in variability. If the IMD value is extremely close to −1 or +1, this shows that there is strong difference in variability between the groups. Furthermore, we used Analysis of Similarity (ANOSIM in Primer v6) analysis, a non-parametric method for estimating differences between groups based on 1,000 random permutations, to compare the bacterial community structures at day 3 between incubated and control nests. ANOSIM was also used to evaluate the bacterial compositional changes over 15 days in incubated eggs or control eggs.

Compositional analysis [75], [76] was used to examine the effect of incubation on the proportion of a taxon in total bacterial abundance. It was conducted at the phylum and separately at the genus level for the dominant taxa. This approach is based on the log-ratio analysis of compositions to overcome problems of proportional data [75], for instance the proportions are dependent on each other in a group and the sum of proportions is one [76], [77]. In the whole set of data (data from incubated and control eggs combined), we chose four most abundant phyla (comprising >90% of all sequences) and in another analysis we chose 10 most abundant genera (comprising >50% of all sequences). Then, for each chosen phylum or genus we divided the proportion of this taxon by the sum of the proportions of all the remaining taxa (i.e. all phyla besides the four major ones, or all the genera besides the 10 chosen ones). We log-transformed the values. If the proportions were zero, we replaced the zeros with 0.0001 [77]. The log-ratio values were analysed using repeated measures MANOVA in SPSS 20.0, to evaluate the interaction effect between incubation treatment (present or absent, i.e. eggs in magpie nest or in control artificial nest) and day of sampling (day 3 or day 18) to determine the effect of incubation on changes in abundance. Then, we separately compared the relative abundance values for each taxon between day 3 and day 18 measurements using paired t-tests. Corrections for multiple comparisons (e.g. Bonferroni correction) have been traditionally applied when comparisons are made on the same set of data. But the use of such corrections has recently been criticized [78], [79] because they may be too conservative. Therefore, following Shawkey et al [12], we used paired t-tests to analyze changes in relative abundance of each genus during our experiment (between day 3 and day 18).

Using the ‘indval’ function in the ‘labdsv’ package in R software [80], we performed a Dufrene-Legendre Indicator Species Analysis [81] to identify bacterial taxa that are strongly indicative of a particular group of samples (either incubated eggs on day 3 or incubated eggs on day 18). Indicator values range from 0 to 1 and provide a measure of the strength of the association between the taxon and the particular group. The Dufrene-Legendre Indicator Species Analysis is based on frequencies of species in a set of samples and therefore is not reliable for very small number of samples. Therefore we did not conduct it for non-incubated eggs.

## Supporting Information

Table S1 **The list of bacterial taxonomy and relative abundance (%).** If a taxon appeared at least in one of 24 samples we used in this study, it was indicated in the list. S1–S9 are from incubated nests and C1–C3 are from non-incubated (control) nests. Each nest was sampled twice at day 3 (before incubation) and day 18 (after incubation). In column A, the taxonomical classification is indicated from phylum, class, order, family, genus, and species name.
(XLSX)
